# Measurement and driving factors of carbon productivity in China’s provinces: From the perspective of embodied carbon emissions

**DOI:** 10.1371/journal.pone.0287842

**Published:** 2023-08-04

**Authors:** Changyi Liang, Peng Gao

**Affiliations:** 1 School of Economics and Management, Southeast University, Nanjing, China; 2 School of Economics, Nanjing University of Posts and Telecommunications, Nanjing, China; University of Galway, Ireland / Anhui University of Finance and Economics, CHINA

## Abstract

Carbon productivity incorporates economic development and carbon emissions within a unified framework for measuring the economic value per unit carbon emissions. In the context of climate change, improving carbon productivity is of great value for promoting low-carbon development in a country or region. From the perspective of embodied carbon emissions, this study constructs an embodied carbon productivity (ECP) index and uses the Logarithmic Mean Divisia Index decomposition method to study the evolution trends and driving factors of ECP in China’s provinces based on *China Interregional Input-Output Tables* for 2002, 2007, 2012, and 2017. The following results were obtained: First, China’s overall ECP showed a continuously increasing trend during the entire period, with the energy efficiency factor playing the largest role among all driving factors. Second, the ECP in 19 of the 30 Chinese provinces continued to increase and the contributions of energy emission ratio, ECP per capita, and population size factors to the increase in ECP presented evident disparities among different provinces. Third, the ECP in three major regions ranged from high to low in the order of East, Central, and West, with the largest growth in the Central, followed by the West, with the smallest in the East. Based on the analysis of research results, we proposed relevant policy recommendations to further improve China’s ECP and achieve low-carbon economy.

## 1. Introduction

With the intensification of climate change and convergence of global economic development, various countries are gradually advocating long-term sustainable development to respond to environmental problems (Yue et al., 2023) [1]. As the world’s largest developing country, China has achieved rapid industrialization and urbanization since the establishment of reform and opening up policies and has realized a large amount of material accumulation. However, economic growth may be considered a major determinant of pollution emissions (Mahmood and Saqib, 2022) [2]. For instance, Yang et al. (2022) [3] found that economic development and progress leads to higher levels of environmental deterioration and pollution. According to the relevant report of International Energy Agency in 2022, China’s carbon emissions increased by 750 million tonnes between 2019 and 2021. China has become one of the top polluting nations in the world (Saqib et al., 2022) [4]. Additionally, the world’s economies have only seven years left to meet their 2030 goals of reducing pollution and curbing global warming (Saqib et al., 2023) [5]. Therefore, China has experienced enormous pressure to reduce its emissions (Song et al., 2021) [6]. To actively address the environmental problems caused by the surge in carbon emissions, the Chinese government has proposed a series of targets and policies to reduce carbon emissions (Sun et al., 2022) [7], among which achieving “peak carbon” by 2030 and “carbon neutrality” by 2060 are the most powerful macro development goals for reducing emissions. In this context, determining methods for improving the quality of China’s low-carbon economy has become an issue that needs to be addressed.

Carbon productivity combines economic growth and carbon emissions and thus can reflect the economic value per unit of carbon emissions; it has been widely used to measure the high-quality development of low-carbon economy. However, in the quantitative analysis of carbon productivity, previous studies have generally only included direct carbon-emission factors, whereas indirect carbon-emission factors generated from the production and consumption of intermediate goods are rarely taken into account; moreover, the obtained carbon productivity often deviates from the actual situation. In addition, with the promulgation of opening up and reform policies and increases in the division of production, the economic linkages among Chinese provinces have become closer and the production chain has been continuously refined, which has led to the segmentation of production and consumption and intensified the flow of carbon emissions among provinces (Mi et al., 2017 [8]; Liu et al., 2020 [9]). Therefore, the objective of this study is to integrate direct and indirect carbon emissions into a unified framework to measure the carbon productivity levels of Chinese provinces from the perspective of embodied carbon emissions. In other words, the embodied carbon productivity (ECP) of Chinese provinces were measured by incorporating embodied carbon emission factors, and further analyzes the driving factors of ECP to support the China’s low-carbon economy and high-quality development.

Compared with the existing studies, the novelty of this study is as follows: First, to calculate carbon productivity, an ECP index is constructed by including the embodied carbon emission factor, which differs from previous studies that only considered direct carbon emissions. Embodied carbon emissions include direct carbon emission factors used in previous studies and indirect carbon emission factors, that have not been thoroughly considered in previous studies. Second, based on the evolutionary characteristics of ECP, this study constructed a factor decomposition model of ECP to study the influence of different factors. An analysis of the drivers clearly showed the factors that have a greater influence on changes in ECP, which will increase the precision of low-carbon development strategies. Third, this study not only analyzed the evolutionary trends and drivers of ECP in each province of China but also divided each province into three major regions (East, Central, and West) and further analyzed the changes in ECP and its influencing factors in these regions. The East, Central, and West regions are divided as follows: the East region includes the 11 provinces (Beijing, Tianjin, Hebei, Liaoning, Shanghai, Jiangsu, Zhejiang, Fujian, Shandong, Guangdong, and Hainan); the Central region includes the 8 provinces (Shanxi, Jilin, Heilongjiang, Anhui, Jiangxi, Henan, Hubei, and Hunan); and the West region includes the 12 provinces (Inner Mongolia, Guangxi, Chongqing, Sichuan, Guizhou, Yunnan, Tibet, Shaanxi, Gansu, Qinghai, Ningxia, and Xinjiang). However, Tibet is not included in this paper due to missing data. As a consequence of this, our work makes contributions to the ongoing research. In addition, the findings of the study will be of assistance to Chinese policymakers in the process of planning and executing policies that are conducive to low-carbon and sustainable development.

The remainder of this paper is organized as follows. Section 2 provides a literature review on carbon productivity and its driving factors. Section 3 constructs a model for measuring and decomposing ECP factors and further explains the data sources. Section 4 analyzes the evolution characteristics and driving factors of ECP in different dimensions. The final section mainly provides conclusions and policy implications for achieving low-carbon economy and improving ECP in China.

## 2. Literature review

With the increasing negative effects of climate change, a worldwide consensus has been reached that the development of low-carbon economy is required. Moreover, the efficiency of low-carbon economic development and curbing climate change have become important issues that need to be solved. Kaya and Yokobori (1997) [10] introduced the concept of carbon productivity and defined it as the ratio of economic growth to carbon emissions. Carbon productivity has been widely used by many scholars as a tool for measuring the quality of low-carbon economy in a country or region. Research on carbon productivity can be summarized based on three specific paths.

First, measurement of carbon productivity. Studies have measured carbon productivity in various dimensions, such as the country, industry sector, and enterprise dimensions (Du and Li, 2019 [11]; Bai et al., 2019 [12]; Yang et al., 2021 [13]). Different methods have been used in these related studies to measure carbon productivity, although three methods are commonly used: the first method uses the Malmquist index of the data envelopment analysis (DEA) model (Zhou et al., 2010 [14]; Pan et al., 2020 [15]); the second method uses stochastic frontier analysis (SFA) (Bai et al., 2019 [12]); and the third method directly uses the ratio of GDP to carbon emissions (Hu and Liu 2016 [16]; Chen et al., 2018a [17]). However, few studies have incorporated carbon emissions into carbon productivity indicators or examined the economic value per unit of carbon emission consumption from an entire life cycle perspective. In literature, only Guo et al. (2021) [18] studied the carbon productivity of Chinese industrial sectors from the perspective of embodied carbon and analyzed the factors influencing carbon productivity; however, their study did not consider variations in carbon productivity by province.

Second, identifying the factors that influence carbon productivity and revealing associated decomposition methods. Many scholars have studied the factors that influence carbon productivity from various perspectives, such as direct carbon emissions, energy consumption, population dynamics, industrial structure, and technological progress (Meng and Niu, 2012 [19]; Pan et al., 2020 [15]; Liu and Zhang, 2021 [20]; Fan et al., 2021 [21]; Guo et al., 2021 [18]). Among China-specific studies, Chen et al. (2018a) [17] investigated the evolution characteristics and drivers of carbon productivity in the Chinese power industry and proposed countermeasures to promote carbon productivity in the power industry from the provincial perspective. Han (2021) [22] investigated the paths of carbon productivity improvement in each Chinese province from a technological innovation perspective. Although the above literature provides scientific support for the study of carbon productivity, it does not include embodied carbon emission factors or consider the perspective of the entire life cycle. In addition, index decomposition analysis, which mainly includes Laspeyres and Divisia decomposition, can quantify the degree of influence of various factors on total emission changes through decomposition and has been widely used in environmental economics studies. Currently, the Logarithmic Mean Divisia Index (LMDI) is considered the best index decomposition method because it can effectively handle zero values and has no residuals (Goh and Ang, 2019 [23]; Dong et al., 2020 [24]; Guo et al., 2021 [18]).

Third, using carbon productivity as a research tool to verify the effectiveness of environmental policies and green technological innovations (Song and Han, 2022 [25]; Meng et al., 2022 [26]), providing an effective solution for the evaluation of low-carbon policies. Gao et al. (2021) [27] investigated the carbon efficiency of Chinese industry sectors from the perspective of embodied carbon emissions and compared direct carbon emissions and embodied carbon emissions as non-desired output conditions in Chinese industry sectors; however, China has unbalanced regional development (Zhang et al., 2022 [28]), they did not consider the differences among Chinese provinces.

The above analysis shows that many scholars have used carbon productivity to study the development of low-carbon economy in a country or region from multiple perspectives. However, there are still some gaps in the existing literature: Firstly, existing studies on carbon productivity have primarily calculated direct carbon emissions and focused less on embodied carbon emissions; therefore, the measured carbon productivity often deviates from the actual situation. Secondly, the existing literature mainly analyzes changes in carbon productivity from the perspective of national or industrial sectors, while few analyses have been performed at the provincial level. Due to the large gaps in the industrial structure and development level among the 30 provinces in China (Song et al., 2021 [6]), it is urgent to reveal the ECP status in different periods at the provincial scale and carry out research on the decomposition of the driving factors of ECP. Finally, existing literature does not consider the impact of trade openness when measuring embodied carbon emissions. In the context of an open economy, trade openness factors are included in this study.

This study aims to close these gaps in existing literature. First, from the perspective of embodied carbon emissions, a non-competitive input-output model and an ECP indicator were constructed by excluding import factors, and the evolution characteristics of the ECP were analyzed. Second, by constructing a factor decomposition model of ECP, the driving factors of ECP were studied from a multidimensional perspective, and the influence of different factors on ECP was clarified. Third, the characteristics and drivers of ECP were analyzed at the provincial and regional level (East, West, and Central), and the relevant characteristics of ECP were assessed in the three regions. In summary, this study contributes to the literature gap by clearly revealing the evolution characteristics and drivers of China’s provincial ECP, and providing insights for achieving the low-carbon economy.

## 3. Methodology and data sources

### 3.1 Embodied carbon productivity index

Based on the basic setup of the input-output theory and with reference to the methodologies of Wang et al. (2020) [29] and Wang and Han (2021) [30], it is assumed that each industry sector *i* in the Chinese provinces satisfies the following conditions:

$$x_{i}=\sum_{j=1}^{n}a_{ij}x_{i}+y_{i}+t_{i}^{e}-t_{i}^{m}(i,j=1,2\cdots ,n)$$ (1)

Extended to *n* industry sectors, the following conversion equation is obtained:

$$\left[\begin{array}{c} X_{1}\\ X_{2}\\ \cdots \\ X_{n} \end{array}\right]={\left[\begin{array}{cccc} I-A_{11} & -A_{12} & \cdots & -A_{1n}\\ -A_{21} & I-A_{22} & \cdots & -A_{2n}\\ \cdots & \cdots & \cdots & \cdots \\ -A_{n1} & -A_{n1} & \cdots & I-A_{nn} \end{array}\right]}^{-1}\left\{[\begin{array}{c} \sum_{i=1}^{n}Y_{1i}\\ \sum_{i=1}^{n}Y_{2i}\\ \cdots \\ \sum_{i=1}^{n}Y_{ni} \end{array}]+[\begin{array}{c} T_{1}^{e}\\ T_{2}^{e}\\ \cdots \\ T_{n}^{e} \end{array}]-[\begin{array}{c} T_{1}^{m}\\ T_{2}^{m}\\ \cdots \\ T_{n}^{m} \end{array}]\right\}$$ (2)

where *X* and *Y* are the total output and final consumption, respectively; *T*^*e*^ and *T*^*m*^ are the export and import value column vectors, respectively; and *A* is the direct consumption coefficient matrix.

Referring to Pu et al. (2020) [31], a non-competitive input-output model was constructed to measure the energy consumption problem by setting the import coefficient matrix *M* and excluding the imported intermediate inputs *A*^*m*^, where the direct consumption coefficient matrix can be expressed as *A* = *A*^*d*^+*A*^*m*^, and *A*^*d*^ is the direct consumption coefficient matrix of domestic inputs.

The direct energy consumption coefficient matrix of the industry sectors in each province of China is set as *E*. Combined with the concept underlying the construction of the input-output model, the indirect energy consumption coefficient matrix of the industry sectors in China can be expressed as follows:

$$W=E[{\left(I-A^{d}\right)}^{-1}-I]$$ (3)

where (*I*−*A*^*d*^)^−1^ denotes the *Leontief inverse matrix*, in which imports are removed from the industry sectors and used to denote intermediate production technology, which measures the participation and production level of each industry sector in the process of producing intermediate goods.

Based on the above analysis, an ECP index was constructed for each province in China by combining the characteristics of carbon productivity.

$$ECP=\frac{VA}{ECEs}$$ (4)

where *ECP* denotes the embodied carbon productivity of each Chinese province and measures the economic value generated per unit of embodied carbon emissions consumed during the entire life cycle of each Chinese province; *VA* denotes the value-added of each province’s industry sector; and *ECEs* denote the embodied carbon emissions of each province’s industry sector.

### 3.2 Factor decomposition model of ECP

According to Hu and Liu (2016) [16], Jia et al. (2017) [32], Chen et al. (2018b) [33], Jia et al. (2018) [34], Chen and Lin (2020) [35], a factor decomposition model for China’s ECP was constructed as follows:

$$ECP=\sum_{i=1}^{n}\frac{Y_{i}}{E_{i}}∙\frac{E_{i}}{{ECE}_{i}}∙\frac{{ECE}_{i}}{Y_{i}}∙\frac{{ECP}_{i}}{P_{i}}∙P_{i}$$

$$={EE}_{i}∙{CE}_{i}∙{CI}_{i}∙{PC}_{i}∙P_{i}$$ (5)

where *P*_*i*_ denotes the population of province *i*, and the remaining variables are consistent with the previous variables; *EE*_*i*_ denotes energy efficiency; *CE*_*i*_ refers to energy consumption per unit of implied carbon, represents the energy emission ratio; *CI*_*i*_ denotes embodied carbon intensity; *PC*_*i*_ denotes ECP per capita; and *P*_*i*_ denotes the population size effect.

The decomposition effects of the various ECP factors in China’s provinces are as follows:

$$\Delta {ECP}_{EE}=\sum_{i=1}^{n}\frac{{ECP}_{i}^{t}-{ECP}_{i}^{0}}{{lnECP}_{i}^{t}-{lnECP}_{i}^{0}}∙ln\left(\frac{{EE}_{i}^{t}}{{EE}_{i}^{0}}\right)$$ (6)

$$\Delta {ECP}_{CE}=\sum_{i=1}^{n}\frac{{ECP}_{i}^{t}-{ECP}_{i}^{0}}{{lnECP}_{i}^{t}-{lnECP}_{i}^{0}}∙ln\left(\frac{{CE}_{i}^{t}}{{CE}_{i}^{0}}\right)$$ (7)

$${\Delta ECP}_{CI}=\sum_{i=1}^{n}\frac{{ECP}_{i}^{t}-{ECP}_{i}^{0}}{{lnECP}_{i}^{t}-{lnECP}_{i}^{0}}∙ln\left(\frac{{CI}_{i}^{t}}{{CI}_{i}^{0}}\right)$$ (8)

$$\Delta {ECP}_{PC}=\sum_{i=1}^{n}\frac{{ECP}_{i}^{t}-{ECP}_{i}^{0}}{{lnECP}_{i}^{t}-{lnECP}_{i}^{0}}∙ln\left(\frac{{PC}_{i}^{t}}{{PC}_{i}^{0}}\right)$$ (9)

$$\Delta {ECP}_{P}=\sum_{i=1}^{n}\frac{{ECP}_{i}^{t}-{ECP}_{i}^{0}}{{lnECP}_{i}^{t}-{lnECP}_{i}^{0}}∙ln\left(\frac{P_{i}^{t}}{P_{i}^{0}}\right)$$ (10)

where Δ*ECP*_*EE*_, Δ*ECP*_*CE*_, Δ*ECP*_*CI*_, Δ*ECP*_*PC*_, and Δ*ECP*_*P*_ represent the drivers of energy efficiency, energy emission ratio, embodied carbon intensity, ECP per capita, and population size on ECP, respectively.

### 3.3 Data source and processing

In this study, we analyzed ECP and its drivers for each province in China by constructing a noncompetitive input–output model and the LMDI decomposition method. Considering the reliability, degree of detail, and uniformity of available data, this study mainly uses the *China Interregional Input-Output Tables* for 2002, 2007, 2012, and 2017. The embodied carbon emissions of each province in China were obtained by adding the embodied carbon emissions of the industrial sectors in each province. Moreover, because of the limited availability of energy consumption data for Tibet, Hong Kong, Macao, and Taiwan, this study selected the remaining 30 provinces in China as research objects.

## 4. Empirical results and analysis

### 4.1 Overall evolution characteristics and driving factors of ECP in China

**Fig 1:**
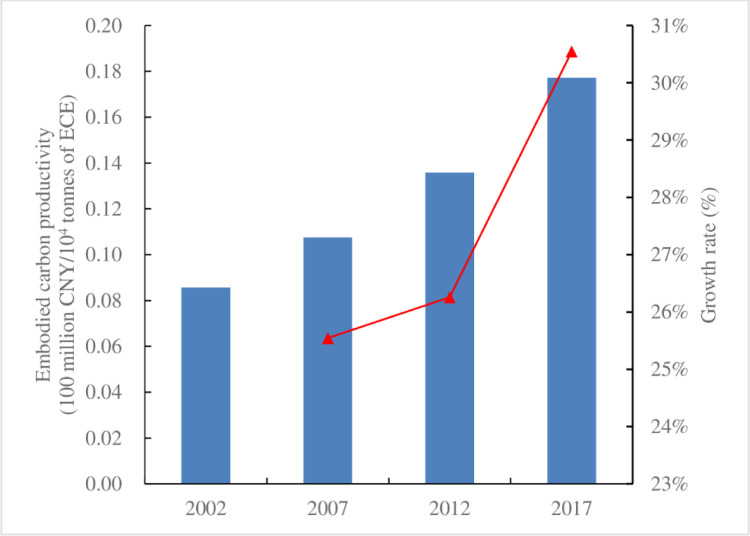
ECP and its growth rate in China during the entire time period. Note: Blue and red columns correspond to the left and right axes, respectively.

China’s overall ECP in 2002, 2007, 2012, and 2017 was 8.57, 10.76, 13.58, and 17.73 million CNY/10^4^ tonnes of ECE, respectively, thus showing a continuous growth trend (Fig 1). This indicates that China’s economic value per unit of increased carbon emissions. In terms of the increase in ECP, China’s overall ECP increased by 9.16 million CNY/10^4^ tonnes of ECE over the entire time period. Our findings on the evolution trend of China’s overall carbon productivity are consistent with Guo et al. (2021) [18], indicating that with the rapid development of China’s ecological civilization and low-carbon economy, its energy efficiency continued to improve, its industrial structure became more optimized, and the rate of energy technology progress gradually accelerated during our study period.

In addition, China’s overall ECP grew by 25.54% in 2007 compared to that in 2002, by 26.26% in 2012 compared to that in 2007, and by 30.54% in 2017 compared to that in 2012. In particular, between 2012 and 2017, China’s overall ECP showed rapid growth, indicating that China’s economic growth rate was much faster than the growth rate of embodied carbon during this period, thus reflecting that China’s economic development is increasingly characterized by low emissions and China is getting closer to achieving its goal of a low-carbon society.

**Fig 2:**
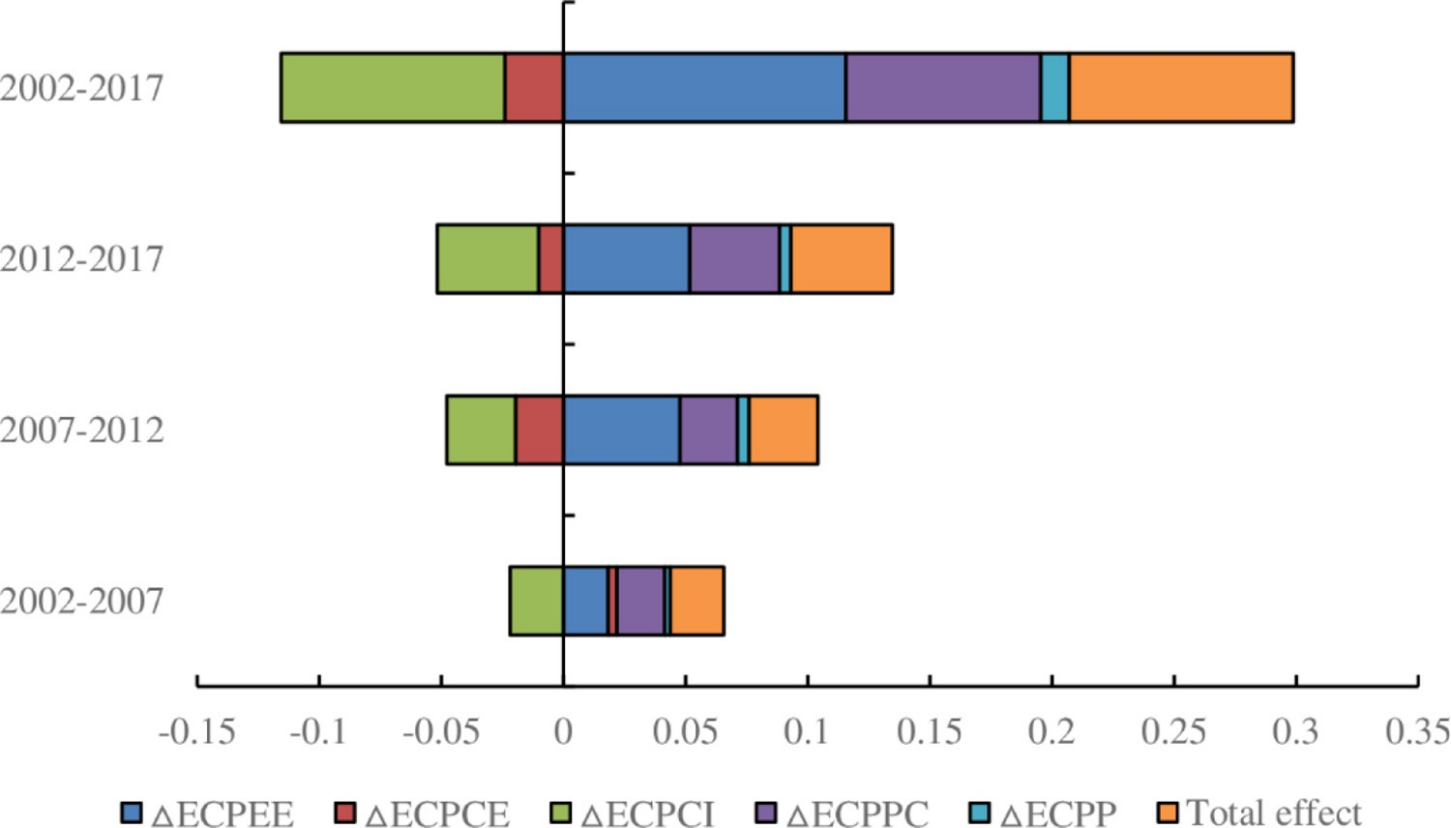
Driving factors of ECP in China. Unit: 100 million CNY/10^4^ tonnes of ECE.

Fig 2 shows the decomposition of the driving factors of ECP in Chinese provinces at different time periods. The contribution value of the total factors of ECP for Chinese provinces is 9.16 million CNY/10^4^ tonnes of ECE over the whole time period, and the contribution value of the total factors of ECP for Chinese provinces is positive and increases gradually over time in all time periods. The contribution value of the total factors of ECP is 2.19 million CNY/10^4^ tonnes of ECE in the period 2002–2007, 2.82 million CNY/10^4^ tonnes of ECE in the period 2007–2012 and 4.15 million CNY/10^4^ tonnes of ECE in the period 2012–2017, indicating that the economic value per unit of carbon emissions has been increasing over time. The decomposition of the drivers of ECP over time shows that the energy efficiency, ECP per capita, and population size factors all contribute to the increase in ECP, with the energy efficiency factor contributing the most. Among these drivers, the contribution value of the energy efficiency factor to the increase in ECP was 11.56 million CNY/10^4^ tonnes of ECE, which was higher than that for the ECP per capita factor (7.99 million CNY/10^4^ tonnes of ECE) and population size factor (1.17 million CNY/10^4^ tonnes of ECE). In addition, Guo et al. (2021) [18] found that energy efficiency factor is the main element affecting change in China’s carbon productivity, which further supports our findings. This shows that it is feasible for the Chinese government to improve energy efficiency to actively respond to climate change and develop the low-carbon economy, as proposed at the *Asia-Pacific Low Carbon Economic Forum China Summit*. Among other driving factors, the energy emission ratio is worthy of attention because it first promotes and then suppresses emissions in different time periods. Specifically, the contribution of the energy emission ratio factor to the increase in ECP was 0.35 million CNY/10^4^ tonnes of ECE in the period 2002–2007; however, it inhibited the increase in ECP over the entire time period because it had a dampening effect during 2007–2012 and 2012–017 at 1.95 million and 1.03 million CNY/10^4^ tonnes of ECE, respectively. The last driver was the embodied carbon intensity factor, which strongly inhibited the increase in ECP between 2002 and 2017 by 9.16 million CNY/10^4^ tonnes of ECE and had a suppressive effect on the increase in ECP in all time periods, with this suppression effect gradually increasing over time. In general, our findings indicate that different driving factors have a promoting or inhibiting effect on the increase in ECP.

The *Carbon Peaking Action Program by 2030* formulated by the Chinese government proposes the “*Top Ten Actions to Achieve Carbon Peak*”, including actions for the low-carbon energy transformation, energy conservation, carbon reduction, and efficiency enhancement. Specifically, vigorously developing new energy, promoting coal consumption substitution and upgrading are all measures that are conducive to improving energy efficiency, reducing carbon intensity and energy emission ratio. These policies have been confirmed to be in line with the long-term evolution trend of China’s ECP. However, the current policies overlook the effects of ECP per capita and population size factors on ECP, and relevant policy recommendations have not been proposed in other studies.

### 4.2 Evolution trend and driving factors of ECP in China’s provinces

Considering the heterogeneity among different provinces in China, the ECP of 30 Chinese provinces in 2002, 2007, 2012, and 2017 were measured and analyzed, and the specific results are shown in Table 1.

**Table 1:** The ECP of 30 provinces in China over the entire time period.

Provinces in China	2002	2007	2012	2017	Provinces’ average value	Total growth rate
Beijing	0.1438	0.1943	0.2094	0.3032	0.2127	110.85%
Tianjin	0.0775	0.1242	0.1516	0.1923	0.1364	148.13%
Hebei	0.0574	0.0538	0.0780	0.0871	0.0691	51.74%
Shanxi	0.0511	0.0349	0.0518	0.0718	0.0524	40.51%
Inner Mongolia	0.0460	0.0639	0.0754	0.0778	0.0658	69.13%
Liaoning	0.0518	0.0782	0.1155	0.0984	0.0860	89.96%
Jilin	0.0808	0.0906	0.1499	0.2301	0.1379	184.78%
Heilongjiang	0.0579	0.1073	0.1461	0.1666	0.1195	187.74%
Shanghai	0.1195	0.1778	0.1871	0.2996	0.1960	150.71%
Jiangsu	0.1234	0.1446	0.2050	0.2949	0.1920	138.98%
Zhejiang	0.1208	0.1456	0.1905	0.2257	0.1707	86.84%
Anhui	0.0896	0.1386	0.1680	0.1809	0.1443	101.90%
Fujian	0.2226	0.1804	0.2471	0.2817	0.2330	26.55%
Jiangxi	0.1239	0.1672	0.1510	0.1662	0.1521	34.14%
Shandong	0.1188	0.0981	0.1153	0.1519	0.1210	27.86%
Henan	0.0919	0.1096	0.1442	0.1775	0.1308	93.14%
Hubei	0.0710	0.1439	0.1691	0.3409	0.1812	380.14%
Hunan	0.1018	0.1284	0.1630	0.2698	0.1658	165.03%
Guangdong	0.1235	0.1856	0.2311	0.2935	0.2084	137.65%
Guangxi	0.1486	0.1404	0.1901	0.2288	0.1770	53.97%
Hainan	0.1473	0.2143	0.2249	0.2237	0.2026	51.87%
Chongqing	0.0754	0.1189	0.1522	0.1875	0.1335	148.67%
Sichuan	0.0677	0.1187	0.1359	0.2599	0.1456	283.90%
Guizhou	0.0325	0.0451	0.0682	0.1115	0.0643	243.08%
Yunnan	0.0729	0.0952	0.1209	0.1859	0.1187	155.01%
Shaanxi	0.0741	0.1066	0.1301	0.1901	0.1252	156.55%
Gansu	0.0308	0.0601	0.0752	0.0970	0.0658	214.94%
Qinghai	0.0344	0.0440	0.0420	0.0402	0.0402	16.86%
Ningxia	0.0261	0.0251	0.0392	0.0376	0.0320	44.06%
Xinjiang	0.0474	0.0876	0.0666	0.0563	0.0645	18.78%
Average value	0.0877	0.1141	0.1398	0.1843	0.1315	120.45%

Unit: 100 million CNY/10^4^ tonnes of ECE.

First, the evolutionary trend of the ECP over the entire period shows that it continued to increase in 19 of 30 Chinese provinces, including Beijing, Tianjin, Inner Mongolia, Jilin, Heilongjiang, Shanghai, Jiangsu, Zhejiang, Anhui, Henan, and some other provinces. This indicates that most of China’s provinces responded positively to the national low-carbon development policy while developing their economies by focusing on controlling carbon emissions and improving carbon productivity. However, there are still some provinces where the ECP has declined over a certain period. Among them, Hebei, Shanxi, Fujian, Shandong, Guangxi, and Ningxia experienced a decline in ECP between 2002 and 2007. Among these provinces, shanxi showed the largest decline, from 5.11 million CNY/10^4^ tonnes of ECE in 2002 to 3.49 million CNY/10^4^ tonnes of ECE in 2007, which represented a decline of 31.70%. Ningxia had the smallest decline in ECP by only 3.83% in 2007 compared to that in 2002. The three provinces of Jiangxi, Qinghai, and Xinjiang experienced a decline in ECP between 2007 and 2012. Among these provinces, Xinjiang’s ECP fell the most, from 8.76 million CNY/10^4^ tonnes of ECE in 2007 to 6.66 million CNY/10^4^ tonnes of ECE in 2012, which represented a decline of 23.97%, while Qinghai’s ECP fell the least, by 4.55%. Between 2012 and 2017, ECP decreased in the five provinces of Liaoning, Hainan, Qinghai, Ningxia, and Xinjiang, with Xinjiang experiencing the largest decrease in ECP, from 6.66 million CNY/10^4^ tonnes of ECE in 2012 to 5.63 million CNY/10^4^ tonnes of ECE, which represented a decrease of 15.47%. Hainan experienced the smallest decrease in ECP, with a decrease of only 0.53% in 2017 compared with that in 2012.

Second, ECP presents evident disparities between different provinces. We compared each province’s ECP with the overall national average ECP over the entire period and found that the ECP values of 16 provinces exceeded China’s ECP average value. From highest to lowest, the provinces are: Fujian, Beijing, Guangdong, Hainan, Shanghai, Jiangsu, Hubei, Guangxi, Zhejiang, Hunan, Jiangxi, Sichuan, Anhui, Jilin, Tianjin, and Chongqing, indicating that these provinces have a relatively leading position in China in terms of ECP. In Fujian, Beijing, and Guangdong, which ranked among the top three over the entire period, the ECP values were 77.19%, 61.77%, and 58.54% above the Chinese average, respectively. The ECP values of the remaining 14 provinces were lower than the Chinese average. From highest to lowest, the provinces are: Henan, Shaanxi, Shandong, Heilongjiang, Yunnan, Liaoning, Hebei, Inner Mongolia, Gansu, Xinjiang, Guizhou, Shanxi, Qinghai, and Ningxia. Taking the bottom three ECP provinces of Ningxia, Qinghai, and Shanxi as examples, their ECP values were lower than the Chinese average by 75.66%, 69.46%, and 60.14%, respectively. Among them, the industries of Shanxi Province, which are rich in coal and other mineral resources, are mainly concentrated in energy industries, such as coal, alumina, coke, steel, electricity, and other high-energy consumption industries. As high energy demand leads to high carbon emissions, Shanxi Province should vigorously adjust its industrial structure, relying on its huge development advantages in energy, to achieve low-carbon economy.

Third, the total ECP growth rate of 14 provinces in China during the entire period was higher than the overall national average value. From highest to lowest, these provinces are: Hubei, Sichuan, Guizhou, Gansu, Heilongjiang, Jilin, Hunan, Shaanxi, Yunnan, Shanghai, Chongqing, Tianjin, Jiangsu, and Guangdong. Hubei, Sichuan, and Guizhou, which ranked among the top three, had total ECP growth rates of 380.14%, 283.90%, and 243.08%, respectively. In contrast, the total ECP growth rates of 16 provinces in China during the entire period were lower than the overall national average. From highest to lowest, these provinces are: Beijing, Anhui, Henan, Liaoning, Zhejiang, Inner Mongolia, Guangxi, Hainan, Hebei, Ningxia, Shanxi, Jiangxi, Shandong, Fujian, Xinjiang, and Qinghai. The total ECP growth rates of Qinghai, Xinjiang, and Fujian, the three provinces with the lowest rankings, were 16.86%, 18.78%, and 26.55%, respectively. Although the total growth rate of Fujian Province was relatively low, its ECP values were high at all time periods in our study, which is different from the situation in Qinghai and Xinjiang.

Currently, the Chinese government proposes that the formulation and implementation of policies in various provinces should be based on the actual economic development and resource endowments of the region. Therefore, policy recommendations to promote the improvement of ECP in different provinces should adapt to local conditions. Specifically, provinces with relatively high ECP should adhere to low-carbon development strategies and further consolidate their achievements. However, since the ECP of some provinces is relatively low in China, it is necessary for these provinces to actively respond to relevant national policies, such as vigorously optimizing industrial and energy structures, in order to gradually narrow the gap in ECP.

The decomposition of the total factors and the contribution values of each driving factor to the ECP in the 30 Chinese provinces over the entire time period are shown in Table 2.

**Table 2:** The driving factors of ECP in China’s provinces over the entire time period.

Provinces in China	Δ*ECP*_*EE*_	Δ*ECP*_*CE*_	Δ*ECP*_*CI*_	Δ*ECP*_*PC*_	Δ*ECP*_*P*_	Δ*ECP*_*Total*_
Hubei	0.2052	0.0647	-0.2699	0.2630	0.0069	0.2699
Sichuan	0.1479	0.0443	-0.1922	0.1891	0.0031	0.1922
Shanghai	0.1984	-0.0183	-0.1801	0.1087	0.0714	0.1801
Jiangsu	0.1784	-0.0070	-0.1714	0.1461	0.0253	0.1714
Guangdong	0.1663	0.0036	-0.1699	0.1076	0.0623	0.1699
Hunan	0.1612	0.0069	-0.1680	0.1679	0.0001	0.1680
Beijing	0.3016	-0.1422	-0.1594	0.0669	0.0925	0.1594
Jilin	0.1769	-0.0276	-0.1493	0.1588	-0.0094	0.1493
Shaanxi	0.1301	-0.0142	-0.1160	0.1081	0.0079	0.1160
Tianjin	0.1491	-0.0343	-0.1148	0.0722	0.0425	0.1148
Yunnan	0.1113	0.0017	-0.1130	0.1034	0.0096	0.1130
Chongqing	0.1318	-0.0197	-0.1122	0.0985	0.0136	0.1122
Heilongjiang	0.0760	0.0328	-0.1087	0.1206	-0.0118	0.1087
Zhejiang	0.1373	-0.0324	-0.1049	0.0619	0.0430	0.1049
Anhui	0.1481	-0.0568	-0.0913	0.0932	-0.0019	0.0913
Henan	0.1324	-0.0468	-0.0856	0.0827	0.0029	0.0856
Guangxi	0.1373	-0.0570	-0.0802	0.0770	0.0032	0.0802
Guizhou	0.0984	-0.0194	-0.0790	0.0796	-0.0006	0.0790
Hainan	0.1256	-0.0492	-0.0764	0.0415	0.0349	0.0764
Gansu	0.0511	0.0152	-0.0662	0.0664	-0.0002	0.0662
Fujian	0.1677	-0.1085	-0.0591	0.0198	0.0393	0.0591
Liaoning	0.0542	-0.0076	-0.0466	0.0447	0.0019	0.0466
Jiangxi	0.1236	-0.0813	-0.0423	0.0328	0.0095	0.0423
Shandong	0.0946	-0.0615	-0.0331	0.0197	0.0134	0.0331
Inner Mongolia	0.0388	-0.0071	-0.0318	0.0305	0.0012	0.0318
Hebei	0.0547	-0.0250	-0.0297	0.0229	0.0068	0.0297
Shanxi	0.0690	-0.0483	-0.0207	0.0168	0.0039	0.0207
Ningxia	0.0209	-0.0094	-0.0115	0.0049	0.0066	0.0115
Xinjiang	0.0176	-0.0086	-0.0090	-0.0047	0.0136	0.0090
Qinghai	0.0233	-0.0175	-0.0058	0.0020	0.0038	0.0058

Unit: 100 million CNY/10^4^ tonnes of ECE.

Note: Sorted by total effect size.

First, the contribution value of the total factors was positive for all Chinese provinces, with an average contribution value of 9.66 million CNY/10^4^ tonnes of ECE. Among them, 14 provinces were above this average value, among which the five provinces with the highest contribution values of the total factors were: Hubei, Sichuan, Shanghai, Jiangsu, and Guangdong, with values of 26.99, 19.22, 18.01, 17.14, and 16.99 million CNY/10^4^ tonnes of ECE, respectively. The bottom five provinces in terms of the contribution value of the total factors were: Qinghai, Xinjiang, Ningxia, Shanxi, and Hebei, and the three provinces with the lowest contribution values were all located in Northwest China.

Second, the contribution value of the energy efficiency factor was positive for all Chinese provinces over the entire period, indicating that the energy efficiency factor had a positive impact on the improvement of ECP in all Chinese provinces. The average contribution value of the energy efficiency factor for all Chinese provinces was 12.10 million CNY/10^4^ tonnes of ECE, with 18 provinces above the average value. From highest to lowest, these provinces were: Beijing, Hubei, Shanghai, Jiangsu, Jilin, Fujian, Guangdong, Hunan, Tianjin, Anhui, Sichuan, Zhejiang, Guangxi, Henan, Chongqing, Shaanxi, Hainan, and Jiangxi. Among them, the contribution values of the energy efficiency factor of the top five provinces were: 30.16, 20.52, 19.84, 17.84, and 17.69 million CNY/10^4^ tonnes of ECE, respectively, which accounted for 29.22% of all provinces in China. Twelve provinces in China are below the average contribution value of the energy efficiency factor, with the lowest to highest contributions being: Xinjiang, Ningxia, Qinghai, Inner Mongolia, Gansu, Liaoning, Hebei, Shanxi, Heilongjiang, Shandong, Guizhou, and Yunnan. The contribution values of the energy efficiency factor for the last five provinces are: 1.76, 2.09, 2.33, 3.88, and 5.11 million CNY/10^4^ tonnes of ECE, respectively, and these five provinces only accounted for 4.18% of the contribution value of all provinces. We found that four of the bottom five provinces in terms of the contribution value of the energy efficiency factor were located in Northwest China, indicating that the contribution value of the energy efficiency factor in the northwest provinces lags behind that in the rest of China.

Third, we analyzed the energy emission factors of ECP for each province in China. During the entire period, the seven provinces: Hubei, Sichuan, Heilongjiang, Gansu, Hunan, Guangdong, and Yunnan had positive energy emission factor contribution values of 6.47, 4.43, 3.28, 1.52, 0.69, 0.36, and 0.17 million CNY/10^4^ tonnes of ECE, respectively. Except for the seven provinces mentioned above, all the other provinces had negative contribution values to the energy emission factor. The five provinces with the lowest contribution value of the energy emissions factor were Beijing, Fujian, Jiangxi, Shandong, and Guangxi, with values of -14.22, -10.85, -8.13, -6.15, and -5.7 million CNY/10^4^ tonnes of ECE, respectively.

Then, we analyzed the embodied carbon intensity factor of the ECP for each province in China. During the entire period, the embodied carbon intensity factor in all provinces was negative, indicating that it had an inhibitory effect on the ECP of all Chinese provinces. The embodied carbon intensity factor of China had an average value of -9.66 million CNY/10^4^ tonnes of ECE, and 17 provinces had values higher than the average value. The top three provinces in terms of the contribution value of the embodied carbon intensity factor were Qinghai, Xinjiang, and Ningxia, all of which are located in the northwestern region, indicating that the embodied carbon intensity factor of the northwestern provinces had a small inhibitory effect on ECP. Thirteen provinces had a lower embodied carbon intensity factor value than the average, with Hubei, Sichuan, and Shanghai presenting the lowest values of -26.99, -19.22, and -18.01 million CNY/10^4^ tonnes of ECE, respectively.

Furthermore, we analyzed the ECP per capita factor for each province in China. There were both positive and negative contribution values of the ECP per capita factor for the Chinese provinces during the entire period. The contribution value of the ECP per capita factor of Xinjiang was -0.47 million CNY/10^4^ tonnes of ECE. Xinjiang was the only province that had a suppressive effect on ECP; however, the suppressive effect is small. All other provinces had a positive contribution to the ECP per capita factor. Among them, the top five provinces were: Hubei, Sichuan, Hunan, Jilin, and Jiangsu in descending order, with values of 26.3, 18.91, 16.79, 15.88, and 14.61 million CNY/10^4^ tonnes of ECE, respectively, indicating that the ECP per capita factor in these provinces has a strong contribution to the increase in ECP. Therefore, it is necessary to propose policy recommendations to reduce the ECP per capita in Xinjiang, in order to weaken its inhibitory effect on ECP.

Finally, we analyzed the population size factor of the ECP in the provinces of China. There are five provinces with negative contribution values of the population size factor. According to the contribution values from low to high, they are Heilongjiang, Jilin, Anhui, Guizhou, and Gansu, with values of -1.18, -0.94, -0.19, -0.06, and -0.02 million CNY/10^4^ tonnes of ECE, respectively. This indicates that policy makers should formulate corresponding policies for these provinces to alleviate the inhibitory effect of population size factor on ECP. In addition, twenty-five provinces presented positive contribution values of the population size factor, and the top five provinces were: Beijing, Shanghai, Guangdong, Zhejiang, and Tianjin, with values of 9.25, 7.14, 6.23, 4.3, and 4.25 million CNY/10^4^ tonnes of ECE, respectively. The above five provinces are relatively developed in China, indicating that the population size factor of these provinces played a significant role in promoting the improvement of ECP.

**Fig 3:**
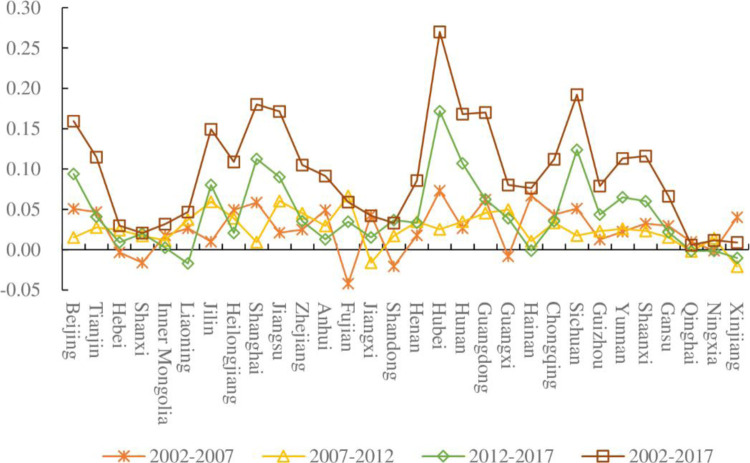
Total effect of ECP in China’s provinces. Unit: 100 million CNY/10^4^ tonnes of ECE.

The factor contribution values of the ECP for the 30 provinces in China over the different sample periods are shown in Fig 3.

First, the following five provinces had negative contribution values of the total factor in the period 2002–2007: Fujian, Shandong, Shanxi, Guangxi, and Hebei, with values of -4.22, -2.07, -1.62, -0.82, and -0.36 million CNY/10^4^ tonnes of ECE, respectively. This indicates that the contribution values of the total factors in these five provinces in the period 2002–2007 decreased over the entire period. In addition, the contribution value of the total factors during the period 2002–2007 was positive for the remaining 25 provinces. Second, the contribution value of the total factors in the period 2007–2012 was negative in Xinjiang, Jiangxi, and Qinghai, with values of -2.1, -1.61, and -0.2 million CNY/10^4^ tonnes of ECE, respectively. The contribution values of all the factors were positive in the remaining 27 provinces. Third, the following five provinces had negative contribution values of the total factors in the period 2012–2017: Liaoning, Xinjiang, Qinghai, and Ningxia, with values of -1.72, -1.03, -0.18, and -0.16 million CNY/10^4^ tonnes of ECE, respectively. Finally, negative contribution values of the total factor in both time periods were observed in Xinjiang and Qinghai, which means that they are the last two provinces among all Chinese provinces in terms of the contribution value of the total factors over the entire period.

### 4.3 Evolution trend and driving factors of ECP in China’s three regions

**Fig 4:**
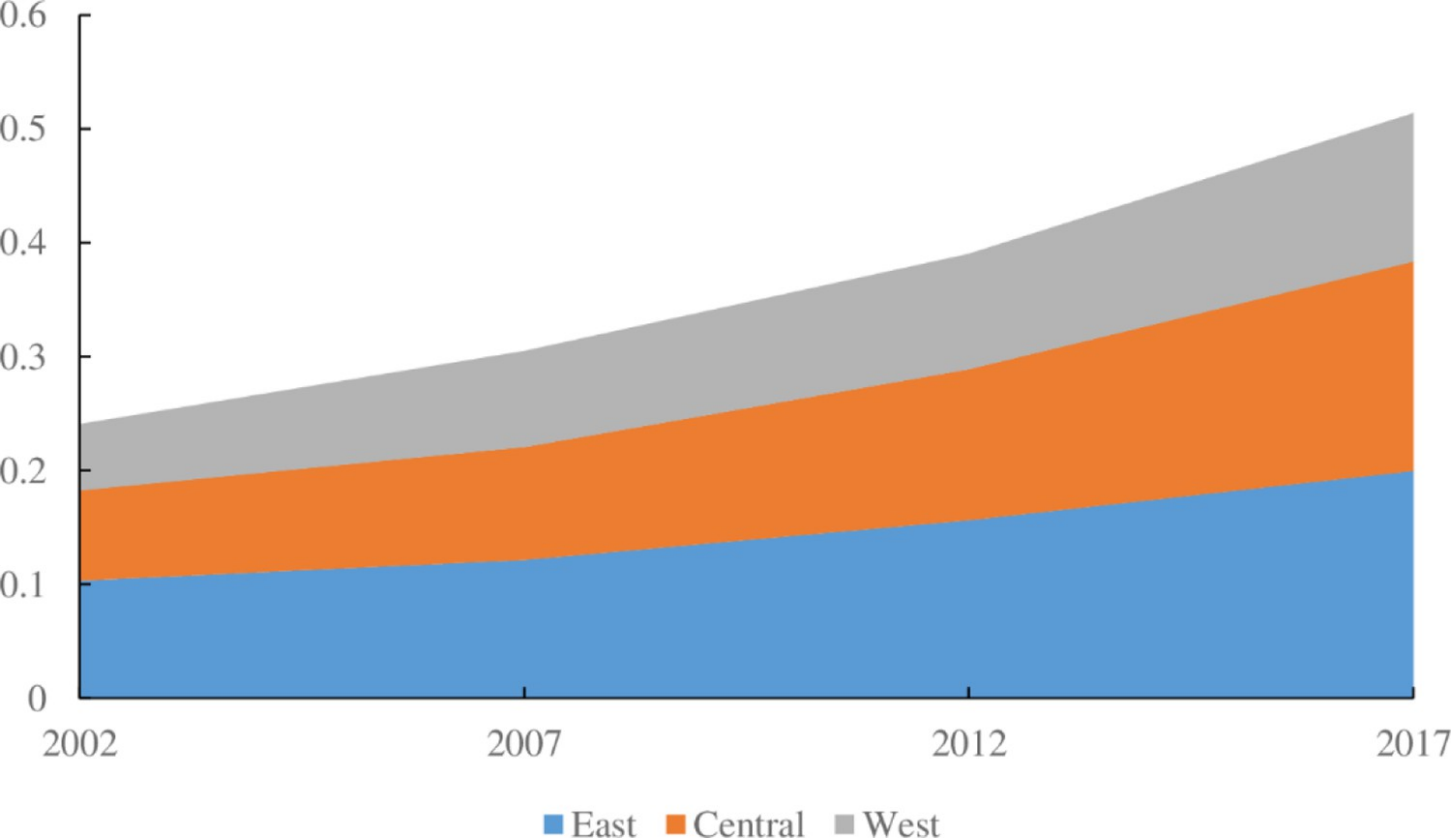
Evolutionary trend of ECP in three regions of China (East, Central, and West). Unit: 100 million CNY/10^4^ tonnes of ECE.

We divided China’s 30 provinces into the three regions East, Central, and West and further analyzed the evolution trend of the ECP in the three regions (Fig 4). Over the entire period, the ECP of all three regions showed a gradual upward trend, with the ECP of the East, Central, and West regions descending in order. The largest increase in ECP was observed in the central region, with an increase of 132.38% in 2017 compared with 2002. The western region was followed by the central region, with an ECP growth rate of 123.65%. The ECP growth rate in the eastern region (93.27%) was the lowest among the three regions. The above results show that although the eastern region’s ECP was already ahead of that of the other regions, this region had less room for improvement, resulting in the lowest growth rate. In contrast, the ECP in the central and western regions has a higher growth rate and greater development potential. In recent years, the economic growth rates of the central and western regions have been higher than those of the eastern region, and the relative gap in regional development continues to narrow. Moreover, consistent trends were observed in the evolution of ECP and economic growth in the three major regions of China in terms of aggregate and regional disparities. Therefore, the eastern region should play a driving force and growth pole role, taking the lead in promoting comprehensive green transformation of economic and social development. The central and western regions should gradually decouple carbon emission growth from economic growth, and strive to further narrow the gap with the eastern region.

The decomposition of the drivers of the ECP in the three major regions of China over different periods is presented in Table 3. During the period 2002–2007, the contribution value of the total factors, from largest to smallest, was West, Central, and East. In the western region, only the embodied carbon intensity factor had a dampening effect on ECP increases. In the central region, energy efficiency and ECP per capita contributed to the increase in ECP. Moreover, the energy emission ratio factor, embodied carbon intensity factor, and population size factor all have inhibitory effects on the increase of ECP. In the eastern region, all drivers contributed to an increase in ECP, except for the embodied carbon intensity factor. During the period 2007–2012, the contribution value of the total factors of the ECP in the East was the highest, followed by the Central, with the lowest values observed in the West. All driving factors had the same effect on the ECP in all three regions. Among them, the energy emission ratio and embodied carbon intensity factors had negative contribution values, whereas energy efficiency, ECP per capita, and population size factors all had positive values. During the period 2012–2017, the contribution value of the total factors was in the descending order of Central, East, and West. The contribution value of each driving factor of the ECP had the same effect direction, which is consistent with the results for the period 2007–2012.

**Table 3:** Driving factors of ECP in three regions of China during different time periods.

Time interval	Three regions	Δ*ECP*_*EE*_	Δ*ECP*_*CE*_	Δ*ECP*_*CI*_	Δ*ECP*_*PC*_	Δ*ECP*_*P*_	Δ*ECP*_*Total*_
2002–2007	East	0.01817	0.00003	-0.01820	0.01161	0.00659	0.01820
	Central	0.02134	-0.00154	-0.01980	0.02031	-0.00051	0.01980
	West	0.01345	0.01282	-0.02627	0.02568	0.00059	0.02627
2007–2012	East	0.05217	-0.01753	-0.03464	0.02366	0.01098	0.03464
	Central	0.05254	-0.01863	-0.03391	0.03260	0.00131	0.03391
	West	0.03874	-0.02205	-0.01670	0.01552	0.00117	0.01670
2012–2017	East	0.06062	-0.01703	-0.04359	0.03507	0.00852	0.04359
	Central	0.05857	-0.00750	-0.05107	0.05091	0.00016	0.05107
	West	0.03198	-0.00300	-0.02898	0.02520	0.00378	0.02898

Unit: 100 million CNY/10^4^ tonnes of ECE.

**Fig 5:**
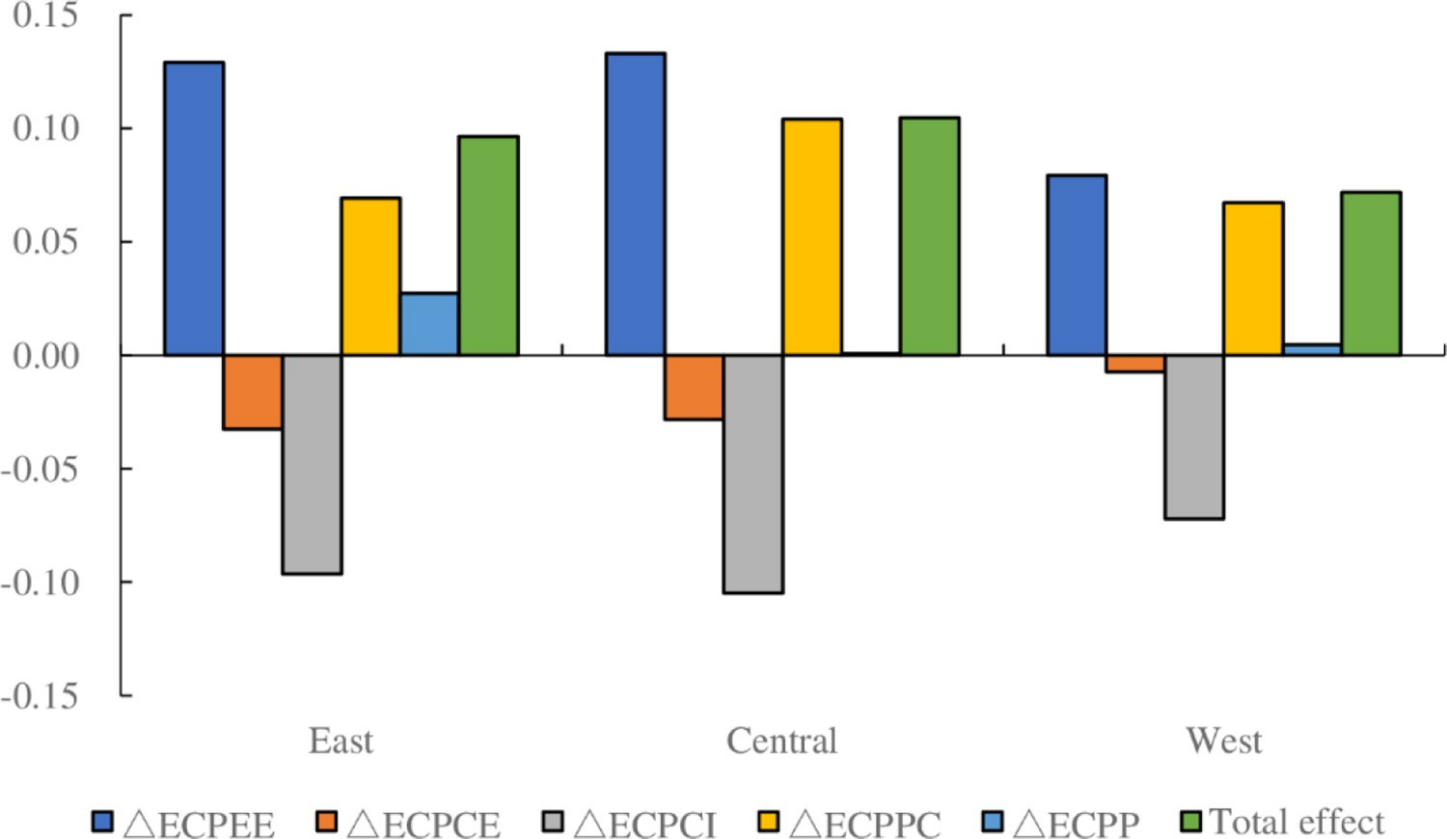
Driving factors of ECP in three regions of China throughout the entire time period. Unit: 100 million CNY/10^4^ tonnes of ECE.

The decomposition of the driving factors of the ECP in the three regions over the entire study period is shown in Fig 5. Among the drivers of ECP in the three regions, the energy emission ratio and embodied carbon intensity factors both had a negative effect on the increase in ECP. Among the two negative driving factors, the energy emission ratio factor had the greatest inhibitory effect on the increase in ECP in the East, followed by the Central, with the smallest observed in the West. We found that the contribution value of energy emission ratio factor in the West was very small, indicating that adopting policies to reduce energy emission ratio might not effectively improve ECP in the West. The embodied carbon intensity factor had the highest to lowest inhibitory effect on the increase in ECP in the Central, East, and West, in descending order. The factors of energy efficiency, ECP per capita, and population size exerted positive effects on the increase in ECP. Among the three positive driving factors, the contribution value of the energy efficiency factor was lower in the West than in the East and Central, while the contribution value of the ECP per capita factor was higher in the central than in the East and West. Although the contribution values of energy efficiency and ECP per capita factors varied among different regions, their promoting effect on the increase of ECP was significant. However, it was most noteworthy that the contribution value of population size factor in the West and Central was very small, significantly lower than that in the East. This indicates that compared to the eastern region, it might be difficult for the western and central regions to achieve significant improvement in ECP by formulating and implementing policies to increase population size.

## 5. Conclusions and policy implications

### 5.1 Conclusions

Based on *China Interregional Input-Output Tables* for 2002, 2007, 2012, and 2017, this study constructed a noncompetitive input–output model to measure the embodied carbon productivity (ECP) index in China, and analyzed the evolutionary characteristics of the province’s ECP. The LMDI decomposition method was then constructed to further analyze the driving factors of the ECP. The findings of our study are as follows: First, the overall ECP showed a continuous growth trend throughout the entire period, especially with a growth rate of 30.54% during the period 2012–2017, and the energy efficiency factor played the largest role among all the drivers of ECP. Second, 19 of China’s 30 provinces consistently improved their ECP over the entire period, and the contribution of each driving factor of ECP, except for energy efficiency and embodied carbon intensity factors, presented evident disparities among different provinces. Third, the ECP of the three major regions from high to low was in the East, Central, and West, and it is worth noting that the contribution values of energy emission ratio and population size factors were very small in the West, while the contribution value of population size factor in the Central are almost zero.

### 5.2 Policy implications

Based on the analysis of the research results, the following policy recommendations are put forward: First, in order to further promote the increase of overall China’s ECP, policy makers should continue to implement policies to improve energy efficiency, reduce energy emission ratio and carbon intensity under the existing policy framework. Specifically, efforts should be made to implement renewable energy substitution and accelerate the construction of a clean and efficient energy system to significantly improve energy utilization efficiency. In addition, it is necessary to strictly control the growth of coal consumption, adjust and optimize industrial and energy structures, research and promote low-carbon technologies, and further reduce energy emission ratio and carbon intensity.

Second, considering that existing studies have not yet paid attention to the effect of ECP per capita and population size factors on ECP, we propose the following corresponding recommendations. On the one hand, given that among all provinces in China, only Xinjiang has a negative effect of the ECP per capita factor on the increase of ECP. At the same time, the driving force of the population size factor in Xinjiang is positive. Therefore, Xinjiang should encourage industrial development to attract more population inflows and better enhance the local ECP. On the other hand, for the five provinces of Heilongjiang, Jilin, Anhui, Guizhou, and Gansu, the effects of ECP per capita and population size factors on the increase of ECP are positive and negative, respectively. Therefore, population migration from these provinces to more developed provinces should be encouraged to reduce the adverse effects on the improvement of local ECP.

Third, the three major regions should have different priorities when formulating relevant policies. Specifically, the eastern region should consider the effect of all driving factors on ECP, while the western region should pay more attention to factors other than energy emission ratio and population size, and the central region should almost ignore population size factor.

Finally, more policy implications relevant to current findings should be provided. For example, it is necessary to further expand the industry coverage of China’s carbon emissions trading market and to apply a more fair and reasonable carbon emissions trading mechanism to reduce greenhouse gas emissions and achieve low-carbon economy.

### 5.3 Future works

This study provides a new approach for measuring carbon productivity in provinces or regions from the perspective of embodied carbon emissions based on data from *China Interregional Input-Output Tables*. Based on our research methodology, other scholars can use datasets from different countries or regions to measure ECP, decompose driving factors, and discuss the constraints and improvement paths of carbon productivity in that country or region to achieve better low-carbon economic development. However, owing to limitations in data availability, the data used in this study were only updated until 2017, resulting in a lack of timeliness, which is the main drawback of this study. We will update the research content of this study once the latest data are available.

## Supporting information

S1 Dataset (XLSX)
